# Family and Spousal Support Are Associated with Higher Levels of Maternal Functioning in a Study of Iranian Postpartum Women

**DOI:** 10.3390/jcm12072718

**Published:** 2023-04-05

**Authors:** Parivash Ahmadpour, Carolann Curry, Shayesteh Jahanfar, Rogaiyeh Nikanfar, Mojgan Mirghafourvand

**Affiliations:** 1 Student Research Committee, Tabriz University of Medical Sciences, Tabriz 5165687386, Iran; 2 Department of Community Medicine, Mercer University School of Medicine, Macon, GA 31207, USA; 3 MPH Program, Department of Public Health and Community Medicine, Tufts University School of Medicine, Boston, MA 02111, USA; 4 Social Determinants of Health Research Center, Department of Midwifery, Tabriz University of Medical Sciences, Tabriz 5165687386, Iran

**Keywords:** family support, spouse support, maternal functioning, postpartum period

## Abstract

Postpartum maternal functioning is a multidimensional concept defined as how a woman manages her daily activities and emotional health after giving birth. This study aimed to determine the predictors of postpartum maternal functioning. This cross-sectional study was conducted on 564 women within one to four months after giving birth (with registered medical records in health centers of Tabriz, Iran) from 2020–2021. The participants were selected based on the cluster sampling method, and data were collected using a standard questionnaire inclusive of sociodemographic and obstetric characteristics, obstetric history, and Barkin Index of Maternal Functioning (BIMF). The adjusted general linear model was employed to estimate the effect of each independent variable (sociodemographic and obstetric characteristics) on the dependent variable (maternal functioning). The mean total score of maternal functioning was 93.1 (±SD = 14.8) out of 120. Based on the adjusted generalized linear model (GLM), “spouse support” and “family support” were strong predictors of maternal functioning. The total score of maternal functioning in women with moderate (B: −4.44; 95% CI: −7.71 to −1.17; *p* < 0.001) and low (B: −4.77; 95% CI: −8.90 to −1.47; *p* < 0.001) spousal support was significantly lower compared to women who received a high level of spousal support. Additionally, this score in women with moderate (B: −5.22; 95% CI: −8.56 to −1.87; *p* < 0.001) and low (B: −3.90; 95% CI: −7.31 to −0.48; *p* < 0.001) family support was significantly lower compared to women who received a high level of family support. Study results suggest that receiving support from both a spouse and family members can improve maternal functioning.

## 1. Introduction

Motherhood is one of the most important roles a woman plays throughout her life [1]. Pregnancy, childbirth, and motherhood can be challenging processes for a woman and her family, both psychologically and socially [2]. As a difficult and potentially traumatic event in life, childbirth may affect a woman’s perception of herself, her ability to accomplish daily activities, and her future opportunities in life [3]. Postpartum recovery is often defined as the recovery of the reproductive organs, but the full recovery of functional abilities is less considered in this regard [4]. Postpartum maternal functioning is a multidimensional concept [5]; Barkin et al. determined that the main domains of maternal functioning during the postpartum period are personal care, infant care, mother–child interaction, maternal psychological well-being, social support, management, and adjustment [6,7]. In general, postpartum maternal functioning gradually improves over time as a woman adjusts to the role [5].

In recent years, the health of mothers and infants after childbirth has received significant attention from researchers [8]. This is due to the fact that postpartum struggles can significantly affect maternal and infant health, infant development, social roles and related coping, personal relationships, and mental disorders. Some of the individual and environmental factors that can affect maternal functioning are the number of previous deliveries the mother has had, quality and adequacy of social and family support, the method of delivery, and complications of pregnancy and delivery [9,10]. Mothers are found to function well during the postpartum period when they receive enough support, balance self care with infant care, and adapt to their various responsibilities [11].

Social support has a positive influence on health during the childbearing period and enables a smooth transition to parenthood. Social support is defined as ‘any informational, tangible, and/or comparison, emotional, a social resource provided to and perceived as effective by the recipient’ [12]. High levels of social capital, especially in low-income communities with poor structural facilities are associated with positive health effects. In different settings, social support during the postpartum period has a positive influence on breastfeeding practices and mothers’ depressive symptoms [13].

Mothers need emotional support after childbirth because it can improve maternal health and promote greater daily functioning [14]. The financial and educational supports provided by spouses, relatives, and health staff can increase self-confidence in new mothers [15]. Webster et al. and Aktan reported a significant relationship between support for mothers and maternal functioning [5,16].

The ability to function well in new motherhood is an important skill and should be assessed in addition to depression/anxiety evaluation. In many settings, primary care services rarely screen for mental health problems during pregnancy or the puerperium. Data from studies in this area indicate the need for further research to explore the effectiveness of efforts to sensitize community health teams to the mental health and maternal functioning challenges women face [17,18]. In this study, factors associated with maternal functioning are explored with an eye towards prevention and maternal/child quality of life.

## 2. Materials and Methods

### 2.1. Study Design and Participants

A descriptive-analytical cross-sectional study was conducted on 564 women visiting health centers of Tabriz, Iran, from 2020–2021. The inclusion criteria were defined as giving birth to only one child within the 1–4 months, following being registered at a maternal clinic. Exclusion criteria were twofold: having a self-reported history of mental illness and failure to complete more than 20% of the questionnaire items.

### 2.2. Sampling

Subsequent to approval from the Ethics Committee of Tabriz University of Medical Sciences, (Ethics code: (Ethics code: IR.TBZMED.REC.1399.246), 564 women who met the inclusion criteria were selected from the health centers of Tabriz based on the cluster sampling method. There are 84 health centers in Tabriz. We first randomly selected a quarter of the health centers (*n* = 21) using www.random.org (accessed on 1 April 2021). Then, researchers provided a list of women who had given birth to a child within the previous one to four months from the established centers. The number of selected samples from each center was determined proportionally based on the number of women referring to each center. Finally, women were randomly selected from the prepared list using www.random.org (accessed on 1 April 2021). The selected women were contacted by telephone and invited to participate in the study. Participants were first briefed on the study objectives and procedure. When the women visited the health center at the specified time, they were again briefed on the study objectives and procedure and asked to sign a written consent form. Women were assured that their information would be kept confidential and the results would be reported anonymously. At that point, participants were provided with questionnaires soliciting information on their sociodemographic and obstetric characteristics. Finally, researchers completed the Barkin Index of Maternal Functioning (BIMF) [19] through interviews with participants.

### 2.3. Data Collection Tool

Sociodemographic and obstetrical characteristics were collected via self-report questionnaire. The Barkin Index of Maternal Functioning [20,21] was administered to capture postpartum maternal functioning.

#### 2.3.1. Sociodemographic and Obstetric Charactristics Questionnaire

This questionnaire consisted of items on age of participants, age of spouse, age at marriage, housing status, educational attainment, spouse’s educational attainment, occupation, spouse’s occupation, body mass index (BMI), spousal support level, family support level, gestational age, parity, history of attending pregnancy classes, and delivery type. This questionnaire was developed by the research team and face/content validity was established.

#### 2.3.2. Barkin Index of Maternal Functioning (BIMF)

We used the BIMF to evaluate the postpartum maternal functioning of study participants. The tool was developed by Barkin et al. in 2007 (Pittsburgh, PA, USA) and consists of 20 items and 7 domains: self-care, infant care, mother-child interaction, psychological well-being, social support, management and adjustment [6,22]. Each item in the BIMF is scored based on a 6-point scale (from 0 to 6), with the total score on this scale ranging between 0 and 120; higher scores indicate better maternal functioning. This index is a patient-centered scale covering all areas of postpartum maternal functioning and can be administered in both clinical and research settings [23]. The psychometric properties of this index have been previously confirmed in Iran [17]. The Iranian version consists of 20 items in 5 subscales: satisfaction with maternal competence, self-care, infant care, social support, and psychological well-being. The Cronbach’s alpha for this study was 0.876 and in line with estimates from various other studies.

### 2.4. Sample Size

The sample size was calculated at 347 with considering the mean score of maternal functioning equal with 97.4 (SD: 13) based on the results of Havizari et al.’s study [24] and α = 0.05, d (precision) = 0.02 around the mean and a test power of 90%. Then assuming a design effect of 1.5 due to the cluster sampling and a possible attrition rate, the final sample size was estimated to be 564.

### 2.5. Data Analysis

Data analysis was performed using SPSS 25 (IBM Inc., Armonk, NY, USA). Participants’ socio-demographic and obstetric characteristics were analyzed using descriptive statistics, including: frequency, percentage, mean, and standard deviation. The results of the Kolmogorov–Smirnov test showed that the quantitative data were following a normal distribution pattern. The relationship of postpartum maternal functioning with sociodemographic and obstetric characteristics was examined using one-way analysis of variance (ANOVA) and the independent *t*-test. Finally, the adjusted general linear model (GLM) was employed to estimate the effect of each of the independent variables (sociodemographic and obstetric characteristics) on the dependent variable (maternal functioning). The results were reported as B coefficient (95% confidence interval). *p* < 0.05 was considered significant.

## 3. Results

The mean age of participants was 29.5 (SD: 6.4) years. More than half (58.1%) had a high school degree or higher and most of them (80.1%) were housewives. The mean age of participant spouses was 33.8 (SD: 6.1) years, more than half of spouses (67%) had a high school degree or higher, and approximately half of them (45.4%) were self-employed. About one-third of participants stated that they were receiving a high level of support from their spouses (39.5%) and family members (32.8%) (Table 1).

The mean total BIMF score of participants was 93.1 (SD: 14.8) (total obtainable range of 0 to 120). The mean score of the BIMF and its subscales is presented in Table 2.

The results of bivariate tests (one-way ANOVA and independent *t*-test) showed that the total score of the BIMF had a significant relationship with housing status (*p* = 0.002), spouse’s occupation (*p* = 0.014), sufficiency of household income (*p* = 0.019), spouse support (*p* < 0.001) and family support (*p* < 0.001), and infant admission to the neonatal intensive care unit (*p* = 0.008). The results of adjusted GLM also indicated that the variables of spouse support, family support, and sufficiency of income for expenses were among the predictors of maternal functioning as the total score of maternal functioning in women with moderate (B: −4.44; 95% CI: −7.71 to −1.17; *p* < 0.001) and low (B: −4.77; 95% CI: −8.90 to −1.47; *p* < 0.001) spouse’s support was significantly lower compared to women who received high level of spouse’s support. Additionally, the score in women with moderate (B: −5.22; 95% CI: −8.56 to −1.87; *p* < 0.001) and low (B: −3.90; 95% CI: −7.31 to −0.48; *p* < 0.001) family support was significantly lower compared to women who received a high level of family support. In addition, the total BIMF score in women with completely sufficient income (B: 4.33; 95% CI: 0.45 to 8.22; *p* < 0.001) was significantly higher compared to women with insufficient income (Table 3).

## 4. Discussion

The results of our study demonstrated that the mean total BIMF score was relatively high in the participants. The results of adjusted GLM also indicated that there was a statistically significant relationship between spouse and family support level and sufficiency of income for expenses and maternal functioning. Notably, the overall BIMF score was higher in participants receiving a high level of support from their spouses and family members, compared to those receiving moderate or low levels of support, as well as in women with completely sufficient income compared to women with insufficient income.

The study results also indicated that the mean maternal functioning score of the participants was 93.1. The mean BIMF score was equal to 97.4 in a study conducted in Iran on 530 primiparous women [24], 96.1 in a study by Williams et al on 46 mothers of neonates admitted to the NICU (Level III) in the Midwestern US [25], and 104 in a study by Barkin et al. on 128 women under obstetric and postnatal care in Georgia [26]. In other researchers by Karami Chamgurdani et al. [17] and Barkin et al. [27] on depressed mothers, the mean BIMF score was low (63.4 in the Karami Chamgurdani et al.’s study and 80.0 in the Barkin et al.’s study). The results indicate that the depressed mothers had the lowest maternal functioning scores. Therefore, health care providers should look for the best solutions to improve maternal functioning of depressed women since it is of great importance for mothers who have recently given birth to a child to optimally play their motherhood role.

In this study, the highest and lowest scores among the subscales of maternal functioning were related to “satisfaction with maternal competence” and “psychological well-being”, respectively. In a study of 305 Iranian primiparous women, the highest and lowest scores among the subscales of maternal functioning within 4 months after delivery were related to “infant care” and “social activities”, respectively [28]. In another study conducted by Mirghafourvand et al. on 165 women, the highest score belonged to “infant care” and “self-care” and the lowest score was related to “social activities” [29]. The above-mentioned two studies employed the Inventory Functional Status after Childbirth (IFSAC) whose subscales are different from those of BIMF. Nevertheless, since the participants in this study gained the lowest score on “psychological well-being”, it is necessary to pay special attention to the mental health of mothers in the postpartum period [30].

The study results suggested that support from both family members and spouses can improve maternal functioning, as family and spouse support is a strong predictor of postpartum maternal health and functioning [31]. In similiar studies, mothers stated that their partners, friends, or even family members can provide key support in infant care, self-care, socialization, and leisure activities [9]. In a study conducted by McVeigh et al. (1995) in Australia, the results indicated that satisfaction with support from family members and friends in infant care positively affected maternal functioning [32]. According to the mothers who participated in a study by Jirapaet et al. (2001) in Thailand, social support had positive effects on maternal functioning [33]. Additionally, a study by Herba et al. (2016) in low- and middle-income countries found that the mental health of mothers and children depends on receiving support [31]. Because of physical changes and fatigue, women need to receive support when they begin taking on maternal responsibilities. Moreover, mothers should be supported both physiologically and psychologically during the postpartum period. Since social support is a strong predictor of maternal functioning, maternal care programs during the pregnancy and postpartum periods should include social support interventions [34].

The results of this study indicated that sufficient income for expenses can lead to enhanced maternal functioning. Receiving social/financial support is a powerful predictor of maternal health and performance after childbirth. In a study conducted by Aktan et al. (2010) in the United States, receiving support was directly related to self-care and social activities under the realm of performance status [5].

Our large sample size (*n* = 564) and the use of a standard, well-validated tool for measuring maternal functioning were two significant strengths of this study. Due to the cross-sectional nature of this study, the relationship shown between spouse and family support and maternal functioning does not necessarily indicate a causal relationship. Therefore, longitudinal and prospective research is needed to establish causality. Another potential limitation of this study is that the level of family and spouse support was measured merely based on one self-report item; future studies are recommended to employ a standard tool for measuring this variable. Ultimately, the findings of this study can help policy makers and providers to develop support programs (emotional, instrumental, and informational) for women during pregnancy and the postpartum period.

## 5. Conclusions

Our study findings suggested a significant relationship between spouse and family support and maternal functioning. The family and spouse support measurement is recommended to be part of pregnancy and postpartum care programs for women in order to identify the those who are receiving low levels of support. Once identified, women receiving low levels of support should receiving training and family counseling sessions with the aim of optimizing maternal functioning.

## Figures and Tables

**Table 1:** Sociodemographic and obstetric characteristics of the participants (*n* = 564).

Characteristics		Relationship with Maternal Functioning	
	Number (Percent)	Mean (SD)	*p*-Value
Age (Year)	29.5 (6.4) *	−0.058 ^¥^	0.736 ^§^
Body mass index (kg/m^2^)	27.5 (3.2) *	0.068 ^¥^	0.149 ^§^
Sufficiency of income for expenses			0.019 ^†^
Completely sufficient	104 (18.4)	75.0 (16.0)	
Somewhat sufficient	326 (57.8)	76.5 (14.5)	
Insufficient	134 (23.8)	81.4 (15.5)	
Home status			<0.002 ^†^
Private house	328 (58.2)	75.5 (14.9)	
corporate home	203 (36.0)	80.8 (14.8)	
Living in a relatives’ house	33 (5.9)	75.3 (16.4)	
Education			0.467 ^†^
Primary school	29 (5.2)	79.5 (18.2)	
Secondary school	96 (17.0)	78.5 (14.8)	
High school	111 (19.7)	81.2 (10.6)	
Diploma	158 (28.0)	77.4 (14.1)	
University	170 (30.1)	73.9 (17.5)	
Spouse education			0.170 ^†^
Illiterate	10 (1.8)	84.9 (13.3)	
Primary school	36 (6.4)	80.5 (17.3)	
Secondary school	66 (11.7)	76.8 (14.3)	
High school	74 (13.1)	80.1 (11.2)	
Diploma	193 (34.2)	78.6 (13.7)	
University	185 (32.8)	74.3 (17.4)	
Job			0.052 ^†^
Housewife	456 (80.9)	78.2 (14.6)	
Working at home	56 (17.0)	74.0 (17.8)	
Working abroad	12 (2.1)	74.5 (10.1)	
Spouse employment			0.014 ^†^
Unemployed	6 (1.1)	86.6 (12.2)	
Government employee	119 (21.1)	73.4 (17.9)	
Manual worker	183 (32.4)	80.2 (14.6)	
Self-employment	256 (45.4)	77.0 (13.7)	
Spouse support			<0.000 ^†^
Low	156 (27.7)	90.0 (14.3)	
Moderate	185 (32.8)	90.4 (14.7)	
High	223 (39.5)	97.5 (14.3)	
Family support			<0.000 ^†^
Low	168 (29.8)	91.1 (15.0)	
Moderate	211 (37.4)	90.5 (14.3)	
High	185 (32.8)	97.8 (14.2)	
Gestational age	37.5 (2.8)		0.534 ^‡^
≤37 (week)	124 (22.0)	77.2 (12.9)	
≥38 (week)	440 (78.0)	77.5 (15.7)	
Para			0.110 ^†^
1	318 (56.4)	93.0 (14.8)	
2	196 (34.8)	94.2 (14.9)	
≥3	50 (8.9)	89.3 (14.3)	
Admission of newborn in NICU			0.008 ^‡^
No	461 (81.7)	92.9 (15.4)	
Yes	103 (18.3)	94.1 (12.2)	
Mode of Delivery			0.253 ^†^
Normal vaginal delivery (NVD)	165 (29.2)	84.6 (10.4)	
Elective cesarean section	178 (31.6)	76.9 (17.0)	
Emergency cesarean section	221 (39.2)	76.7 (13.2)	

* The numbers show mean (standard deviation); ^†^ One-way ANOVA; ^‡^ Independent *t*-test; ^§^ Pearson Correlation test; ^¥^ Correlation coefficient.

**Table 2:** The status of maternal functioning and subdomains (*n* = 564).

Variable	Mean (SD) *	Obtained Score Range	Obtainable Score Range
Maternal functioning	93.1 (14.8)	33 to 120	0 to 120
Satisfaction with Maternal Competence	38.6 (6.7)	15 to 48	0 to 54
Self-care	18.4 (3.4)	5 to 24	0 to 24
Infant care	9.9 (1.9)	2 to 12	0 to 12
Social Support	14.3 (3.3)	1 to 18	0 to 18
Psychological Well-being	4.5 (3.3)	0 to 12	0 to 12

* Mean (standard deviation).

**Table 3:** Predictors of maternal function based on general linear model (*n* = 564).

Variable	B (CI 95% *)	*p*-Value
Spouse support (Reference: High)		
Moderate	−4.77 (−7.58 to −1.08)	0.005
Low	−4.44 (−6.80 to 0.07)	0.008
Family support (Reference: High)		
Moderate	−3.90 (−7.31 to −0.48)	0.025
Low	−5.22 (−8.58 to −1.87)	0.002
Spouse employment (Reference: Self-employment)		
Unemployed	−5.10 (−16.79 to 6.68)	0.398
Employee	1.37 (−1.81 to 4.56)	0.397
Manual worker	−1.45 (−4.20 to 1.30)	0.299
Home status (Reference: Living in a relatives’ house)		
Private house	4.06 (−1.24 to 9.37)	0.133
corporate home	1.08 (−4.36 to 6.53)	0.695
Sufficiency of income for expenses (Reference: Insufficient)		
Completely sufficient	4.33 (0.45 to 8.22)	0.029
Somewhat sufficient	2.26 (−0.78 to 5.30)	0.146
Admission of newborn in NICU (Reference: Yes)		
No	−1.61 (−1.00 to 1.61)	0.318

* Confidence Interval 95%.

## Data Availability

If required, our data can be submitted.
